# Lifespan Trajectories of Asymmetry in White Matter Tracts

**DOI:** 10.1101/2025.09.29.678806

**Published:** 2025-09-29

**Authors:** Praitayini Kanakaraj, Sam Bogdanov, Michael E. Kim, Jessica Samir, Chenyu Gao, Karthik Ramadass, Gaurav Rudravaram, Nancy R. Newlin, Derek Archer, Timothy J. Hohman, Angela L. Jefferson, Victoria L. Morgan, Alexandra Roche, Dario J. Englot, Susan M. Resnick, Lori L. Beason Held, Laurie Cutting, Laura A. Barquero, Micah A. D’Archangel, Tin Q. Nguyen, Kathryn L. Humphreys, Yanbin Niu, Sophia Vinci-Booher, Carissa J. Cascio, The HABS-HD Study Team, Alzheimer’s Disease Neuroimaging Initiative, The BIOCARD Study Team, Zhiyuan Li, Simon N. Vandekar, Panpan Zhang, John C. Gore, Stephanie J. Forkel, Bennett A. Landman, Kurt G. Schilling

**Affiliations:** 1 Vanderbilt University, Department of Computer Science, Nashville, TN, USA; 2 Vanderbilt University, Medical Scientist Training Program, Nashville, TN, USA; 3 Department of Psychological Science and Neuroscience, Belmont University, Nashville, TN, USA; 4 Vanderbilt University, Department of Electrical and Computer Engineering, Nashville, TN, USA; 5 Vanderbilt University Medical Center, Vanderbilt Memory and Alzheimer’s Center, Nashville, TN, USA; 6 Vanderbilt University Medical Center, Vanderbilt Genetics Institute, Nashville, TN, USA; 7 Vanderbilt University, Vanderbilt Brain Institute, Nashville, TN, USA; 8 Vanderbilt University Medical Center, Department of Medicine, Nashville, TN, USA; 9 Vanderbilt University Medical Center, Department of Neurology, Nashville, TN, USA; 10 Vanderbilt University, Department of Psychology and Human Development, Nashville, TN, USA; 11 Vanderbilt University Medical Center, Department of Psychiatry and Behavioral Sciences, Nashville, TN, USA; 12 Vanderbilt University Medical Center, Department of Radiology and Radiological Sciences, Nashville, TN, USA; 13 Vanderbilt University Institute of Imaging Science, Nashville, TN, USA; 14 Vanderbilt University, Department of Biomedical Engineering, Nashville, TN, USA; 15 Vanderbilt University Medical Center, Department of Neurological Surgery, Nashville, TN, USA; 16 Laboratory of Behavioral Neuroscience, National Institute on Aging, National Institutes of Health, Baltimore, MD, USA; 17 Peabody College of Education and Human Development, Department of Special Education, Nashville, Tennessee, USA; 18 University of Kansas, Life Span Institute and Department of Psychology, Lawrence, KS, USA; 19 Department of Computer Science, Park University, Parkville, MO, USA; 20 Vanderbilt University Medical Center, Department of Biostatistics, Nashville, TN, USA; 21 Donders Centre for Cognition, Radboud University, Nijmegen, the Netherlands; 22 Max Planck Institute for Psycholinguistics, Nijmegen, the Netherlands; 23 Brain Connectivity and Behaviour Laboratory, Paris, France

**Keywords:** White Matter, Asymmetry, Diffusion MRI, Lateralization, Lifespan Development, Tractography

## Abstract

Asymmetry in white matter is believed to give rise to the brain’s capacity for specialized processing and is involved in the lateralization of various cognitive processes, such as language and visuo-spatial reasoning. Although studies of white matter asymmetry have been previously documented, they have often been constrained by limited age ranges, sample sizes, or the scope of the tracts and structural features examined. While normative lifespan charts for brain structures are emerging, comprehensive charts detailing white matter asymmetries across numerous pathways and diverse structural measures have been notably absent.

This study addresses this gap by leveraging a large-scale dataset of 26,199 typically developing and aging individuals, ranging from 0 to 100 years of age, from 42 primary neuroimaging studies. We generated comprehensive lifespan trajectories for 30 lateralized association and projection white matter tracts, examining 14 distinct microstructural and macrostructural features of these pathways.

Our findings reveal that: (1) asymmetries are widespread across the brain’s white matter and are present in all 30 pathways; (2) for a given pathway, the degree and direction of asymmetry differ between features of tissue microstructure and pathway macrostructure; (3) asymmetries vary across and within pathway types (association and projection tracts); and (4) these asymmetries are not static, following unique trajectories across the lifespan, with distinct changes during development, and a general trend of becoming more asymmetric with increasing age (particularly in later adulthood) across pathways.

This study represents the most extensive characterization of white matter asymmetry across the lifespan to date, charting how lateralization patterns emerge, mature, and change throughout life. It provides a foundational resource for understanding the principles of white matter organization from early to late life, its relation to functional specialization and inter-individual variability, and offers a key reference for interpreting deviations during healthy development and aging as well as those associated with clinical populations.

## Introduction

Brain asymmetry is an organizing principle of the nervous system that enables functional specialization.^1^ Nearly two centuries ago, the pioneering clinical observations of Marc Dax (1836)^2,3^ and later Paul Broca (1860s)^4,5^ first linked left-hemisphere lesions to specific language deficits, establishing that complex cognitive functions are frequently lateralized to one hemisphere. This hemispheric division of labor is supported by an underlying structural architecture that enhances neural efficiency, allowing for parallel processing of different computations while minimizing cross-hemispheric conduction delays.^6^ While asymmetries in functions as diverse as language,^7^ visuospatial attention,^8^ and motor control^9^ are thought to depend on this lateralized brain circuitry, a comprehensive map of the anatomical scaffold (i.e., the white matter pathways) that supports these functions^10^ across the human lifespan has yet to be charted.

The structural substrate of this functional specialization is the brain’s white matter connectome.^11,12^ Modern neuroimaging - specifically, diffusion-weighted MRI (dMRI) in combination with fiber tractography^13^ - enables a noninvasive “virtual dissection” to segment and study the macrostructure of these pathways, as well as a “virtual histology” to probe their tissue microstructure.^14^ Macrostructural measures describe morphometric features of pathways size and geometry including volume, lengths, and areas.^15^ In contrast, microstructural measures are sensitive to cellular-level features including axonal coherence, packing density, and myelination.^16^ Characterizing white matter asymmetry at both macro- and microstructure scales is essential, as they capture distinct biological properties that can follow different trajectories across development and aging.

Despite steady progress,^17–27^ current knowledge of white-matter asymmetry rests largely on static snapshots from studies with modest sample sizes, narrow age ranges, and limited pathway and feature coverage. This has led to a fragmented literature in which the reported laterality for a given pathway often diverges, due to variations in sample size, age range/cohorts, and the specific structural feature(s) investigated. Moreover, most reports simply focus on testing whether the group mean differs from zero, rather than describing the age-specific distribution and how it changes across development and aging. This leaves unanswered questions about when asymmetry emerges, whether it strengthens or weakens at different life stages, and how widely it varies among healthy individuals at a given age. In short, the field lacks a lifespan-wide normative reference that quantifies the distribution of tract-level asymmetry across development and aging.

Here, we assemble 26,199 scans from 42 population-based cohorts spanning 0–100 years and construct age-varying brain asymmetry charts for white-matter pathways. Rather than centering on a binary test of whether asymmetry exists, we use an estimation framework to quantify the *magnitude*, *direction*, and *age-specific distribution* of asymmetry. Specifically, we: (1) establish normative lifespan trajectories for all investigated features and pathways; (2) identify which tracts and features exhibit asymmetry during key periods of development, young adulthood, and aging; (3) quantify the population-level distribution of right–left asymmetries within these periods; (4) determine whether - and at what ages - the direction of asymmetry reverses; and (5) assess how the magnitude of asymmetry changes dynamically (i.e., increases or decreases) across infancy, development, and later life. Together, these charts provide a population reference to contextualize individuals and cohorts, enabling future developmental and clinical studies to interpret asymmetry in an age-appropriate framework.

## Materials and Methods

**Figure 1.**
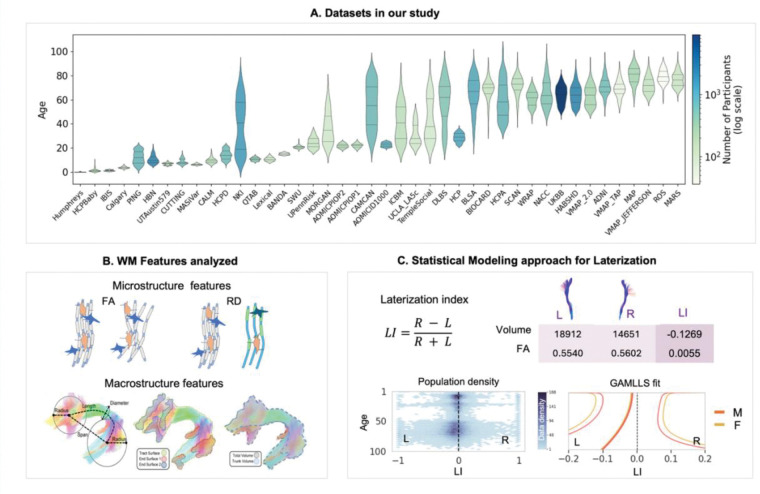
Overview of the study datasets, white matter features, and analytical framework. (**A**) Age distributions for each of the 42 contributing datasets (violin plots), illustrating broad coverage from 0–100 years. Color encodes the number of participants per dataset (log scale). (**B**) Features extracted for each of the 30 bilateral pathways. Microstructural indices (e.g., Fractional Anisotropy, and Mean, Axial and Radial diffusivities; FA, MD, AD, RD) summarize tissue organization and axonal/myelin density; macrostructural indices (e.g., tract volume, length, surface area) capture pathway size and geometry. (**C**) Analysis pipeline. For each participant, white matter pathways were segmented, and features were extracted. A Lateralization Index (LI) was calculated for each tract-feature pair. These LIs were used as input for a normative modeling framework (GAMLSS) to generate age-specific population centile curves.

We analyzed 26,199 diffusion MRI scans from 42 population-based cohorts spanning 0–100 years (Figure 1A). For each participant, we derived tract-level measures for 30 bilateral white-matter pathways, capturing both microstructure (diffusion tensor imaging indices reflecting tissue organization, axonal density, and myelination) and macrostructure (tract volume, length, surface area) (Figure 1B). We computed a laterality index for every tract-feature pair (Figure 1C) and modelled age-varying centile curves to estimate the population distribution (i.e. typically showing 2.5th-25th-50th-75th-97.5th centiles) at each age. Our analyses summarized patterns across the developmental and aging windows, quantify inter-individual variability, and identify tracts and features with asymmetry or direction reversals.

We note that we emphasize and describe population *centiles* - which describe the expected range of individual values at a given age - rather than *confidence intervals*, which only quantify the precision of the median estimate; we therefore do not perform null-hypothesis significance tests, which are trivially positive at very large N. Study cohorts, image preprocessing, white matter pathway segmentation, statistical analysis and normative modeling are described below.

### Participants and Study Cohorts

The final dataset comprised 26,199 cross-sectional diffusion MRI scans aggregated from 42 independent, population-based cohorts (Figure 1A; see Supplementary Table 1 for a detailed description of each cohort). To ensure a cross-sectional analysis, only the earliest available scan was included for any participant with longitudinal data, so that each individual is represented only once. The cohorts consist of typically developing and aging participants with broad geographic representation. Demographic information was harmonized across studies where available, including sex (44.4% female) and handedness (of 5,553 participants with handedness data, 10.6% were left-handed and 0.9% were ambidextrous). Cognitive and behavioral measures were not consistently available across the consortium and were therefore not included in the present analysis. All contributing studies received ethical approval from their local institutional review boards.

### Diffusion MRI Processing Pipeline

All diffusion MRI data were processed with a single, standardized workflow using the PreQual pipeline (v 1.0.8) applied identically across cohorts to promote reproducibility.^28^ This pipeline corrected for susceptibility-induced EPI distortions, head motion, and eddy current artifacts. To ensure consistency and reproducibility across the 42 cohorts, which varied in scanner and acquisition parameters, the same pipeline version was applied to all datasets, with specific flags adapted for each study’s raw data structure. Following this preprocessing, diffusion tensor imaging (DTI) models were fitted to generate voxel-wise maps of fractional anisotropy (FA), mean diffusivity (MD), axial diffusivity (AD), and radial diffusivity (RD). Quality control was conducted at two stages.^29,30^ First, automated logs and visual reports from PreQual were reviewed to identify corruption, gross motion, or failed corrections. Second, the derived scalar maps were visually inspected to verify anatomical plausibility and artifact mitigation. Scans failing QC at either stage were excluded prior to downstream analyses.

### White Matter Pathway Segmentation and Feature Extraction

#### Tract Segmentation

The preprocessed dMRI data for each participant was upsampled to 1 mm isotropic resolution and input into TractSeg (v2.8),^31^ a convolutional neural network (CNN) framework for white matter bundle segmentation.^31^ TractSeg operates directly on local fiber orientation images, segmenting tracts without requiring whole-brain tractography or inter-subject registration. The pre-trained model automatically segmented 72 white matter bundle per participant. From these, we selected 60 lateral pathways (i.e., 30 bilateral tract pairs), excluding midline commissural structures, for asymmetry analyses. The set spans association and projection systems (with limbic, thalamic, and striatal subdivisions noted in Table 1).

#### Microstructural and Macrostructural Feature Definition

For each of the 30 segmented tract pairs, we computed two classes of features using the scilpy library (Sherbrooke Connectivity Imaging Lab’s open-source toolkit, version 1.5.0)^32^ (Figure 1B).

##### Microstructural Features:

We calculated four standard DTI indices that reflect cellular-level tissue properties.^16,33^ To derive a single value for each tract, we computed a weighted average of the metric across all voxels within the tract’s mask, with the contribution of each voxel weighted by the number of streamlines passing through it.

- Fractional Anisotropy (FA): an index of directional coherence of diffusion, influenced by axonal organization/packing and myelin sheaths.
- Mean Diffusivity (MD): the average magnitude of water diffusion, sensitive to overall water mobility within tissue, influenced by axonal/myelin densities.
- Axial Diffusivity (AD): magnitude of water diffusion parallel to the principal fiber direction sensitive to the intra-axonal space and changes in axonal caliber and architecture.
- Radial Diffusivity (RD): magnitude of water diffusion perpendicular to the principal fiber direction. As myelin sheaths are a primary barrier to perpendicular diffusion, increased RD is often interpreted as reflecting demyelination or reduced axonal packing.

##### Macrostructural Features:

We calculated three features describing the large-scale geometry and morphology of each tract^15,34^:

- Tract Volume: The total volume in mm^3^ occupied by the pathway.
- Mean Streamline Length: The average length in mm of the streamlines constituting the tract, reflecting the trajectory extent.
- Tract Surface Area: The surface area in mm^2^ of the geometric shape enclosing the tract, indexing bundle envelope size/complexity.

### Statistical Analysis and Normative Modeling

#### Calculation of the Lateralization Index (LI)

For each micro- and macrostructural feature, we calculated a *Lateralization Index* (LI) to quantify the degree of asymmetry between the left (L) and right (R) hemispheres for each tract (Figure 1C). We used the formula $LI=\frac{R\;-\;L}{R\;+\;L}$ This index is bounded between −1 and 1. An LI of 0 indicates perfect symmetry. For this study, positive LI values indicate a rightward asymmetry (i.e., a higher value in the right-hemisphere tract), while negative LI values indicate a leftward asymmetry (a higher value in the left-hemisphere tract).^35^

#### Lifespan Normative Modeling Framework

We created lifespan charts of white matter asymmetry using a normative modeling approach. In the context of this large-scale dataset (N > 26,000), traditional null-hypothesis significance testing is not informative, as even biologically negligible asymmetries would yield a statistically significant result. Our goal was therefore not to ask the binary question of *whether* asymmetry exists, but rather to adopt an estimation framework to describe the full, age-specific distribution of the LI. This approach, analogous to the creation of pediatric growth charts,^36,37^ allows for the characterization of the typical range of inter-individual variation at any given age.^38^

To model these complex, non-linear lifespan trajectories, we employed Generalized Additive Models for Location, Scale, and Shape (GAMLSS), implemented in R using the gamlss package.^38,39^ GAMLSS is highly suited for lifespan data as it flexibly models how the entire distribution of the LI - including its median (location, μ), variability (scale, σ), and shape - changes as a non-linear function of age.

For each of the 30 tracts and 7 features, a separate GAMLSS model was fit. We modeled the LI as a response variable following a normal distribution,^35^ where both the mean (μ) and the standard deviation (σ) were modeled as functions of age, sex, and study cohort:

(1) $$\mu =f_{\mu }(\mathrm{Age})+\beta_{\mu ,\mathrm{sex}}\cdot \mathrm{Sex}+u_{\mu ,\mathrm{dataset}}$$

(2) $$log(\sigma )=f_{\sigma }(\mathrm{Age})+\beta_{\sigma ,\mathrm{sex}}\cdot \mathrm{Sex}+u_{\sigma ,\mathrm{dataset}}$$

Here, fμ(⋅) and fσ(⋅) are smooth functions of age, Sex is a fixed effect, and dataset random effects ($u_{\mu },u_{\sigma }$) capture residual between-study variation. Allowing σ to vary with age accommodates age-dependent heteroscedasticity.

Age effects were represented with fractional polynomial (FP) smooths (order up to 2; powers drawn from [−2, −1, −0.5, 0, 0.5, 1, 2, 3], with logarithmic terms for repeated powers). For each tract–feature we fit candidate models with different FP choices for μ and σ and selected the specification with the lowest Bayesian Information Criterion (BIC).^40^

From the parameters of the best-fitting model, we generated smooth centile curves (e.g., 2.5th, 25th, 50th, 75th, and 97.5th percentiles) across a dense age grid, forming the final normative brain asymmetry charts (Figure 1C).

#### Characterizing Lifespan Dynamics

To examine white matter asymmetry across the lifespan, we used the normative LI trajectories derived from our GAMLSS models to address four core research questions:

1. What are the characteristics of exemplar lifespan asymmetry curves?

To answer this, we visualized the full, sex-specific centile curves (2.5th to 97.5th percentile) for selected white matter tracts and features to illustrate general patterns of development, aging, and inter-individual variability.

- Which features and pathways exhibit population-level asymmetry at critical lifespan stages?

We summarized tract- and feature-specific asymmetries at four representative ages − 3, 12, 30, and 50 years - using heatmaps that display both the population median LI and the proportion of individuals with rightward vs. leftward lateralization.

- Do asymmetries reverse direction across the lifespan?

We identified shifts in hemispheric dominance by assessing zero-crossings in the median LI trajectories, which indicate if and when the direction of population-level asymmetry changed (e.g., from leftward to rightward, or vice versa).

- How does the magnitude of asymmetry evolve during different life stages?

To answer this, we quantified the rate of change in asymmetry within four distinct age windows: early/late childhood (2–12 years), adolescence (12–20 years), young adulthood (20–40 years), and middle/late adulthood (40–100 years). Within each window, we calculated the mean slope of the absolute value of the median LI curve to determine whether the *magnitude of asymmetry* was increasing (strengthening) or decreasing (attenuating) during that life stage.

## Results

We constructed normative lifespan charts of white matter asymmetry by applying our statistical modeling framework to 26,199 individuals spanning 0–100 years. These charts characterize the magnitude, direction, and population-level variability for 7 micro- and macrostructural features across 30 bilateral white matter pathways. This comprehensive analysis revealed several key principles of brain lateralization, which are detailed in the following sections. We begin by illustrating the modeling approach and showing exemplar trajectories that highlight distinct patterns of development and aging.

### What are the characteristics of exemplar lifespan asymmetry curves?

**Figure 2.**
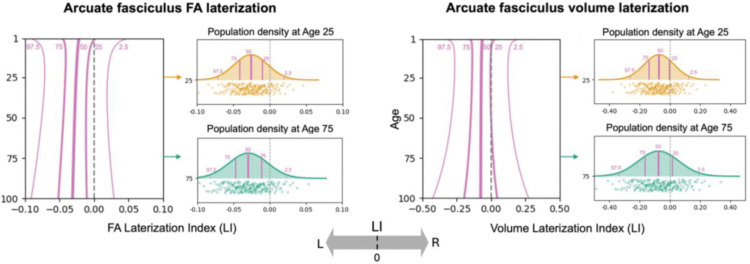
Exemplar asymmetry lifespan curves for the arcuate fasciculus (AF). Lifespan trajectories for fractional anisotropy (FA; a microstructural feature) and tract volume (a macrostructural feature) are shown. Lines represent the 2.5th, 25th, 50th (median), 75th, and 97.5th centile curves, illustrating the full population distribution of the Laterality Index (LI) across the lifespan. Negative values indicate leftward asymmetry, positive values indicate rightward asymmetry.

The normative lifespan charts revealed that white matter tracts exhibit highly heterogeneous and dynamic patterns of asymmetry, which varied substantially across different pathways, features, and ages. To orient the reader to these results, Figure 2 illustrates the full centile-based lifespan trajectory for two features of the arcuate fasciculus (AF) – the FA and total volume. These charts depict the population median (50th percentile) and the spread of the population (from the 2.5th to the 97.5th percentile), with lateralization intuitively shown left-to-right along the x-axis, providing a comprehensive view of both the central tendency and the inter-individual variability in asymmetry at any given age.

**Figure 3.**
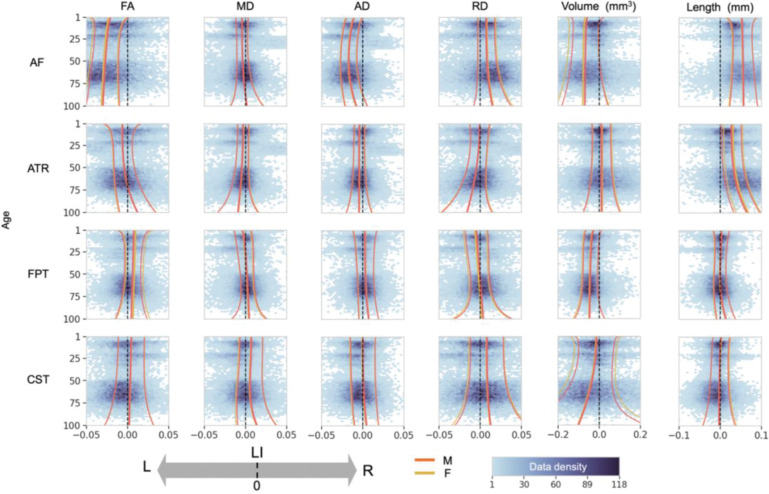
Diverse patterns of lifespan asymmetry across tracts and features. Lifespan trajectories of the LI are shown for four white matter tracts (arcuate fasciculus (AF), anterior thalamic radiation (ATR), frontal pontine tract (FPT), and corticospinal tract (CST)) and six features (FA, MD, AD, RD, volume, and length). Solid lines represent the population median (50th percentile), with the lighter bands showing the inter-quartile range (25th-75th percentiles). Red and yellow lines correspond to males and females, respectively. The plots highlight that the magnitude, direction, and age-related dynamics of asymmetry are highly specific to the tract and feature being measured. Full charts for all tracts and features are provided in Supplementary Material (Supplementary Fig. S2).

Building on this, Figure 3 displays a broader selection of exemplar trajectories for four well-studied pathways (AF, Anterior Thalamic Radiation [ATR], Frontal Pontine Tract [FPT], Corticospinal Tract [CST]) across six key micro- and macrostructural (FA, MD, AD, RD, tract volume, tract length) features. These charts highlight several overarching principles.

First, while asymmetry is common, its magnitude varies by feature type. Microstructural asymmetries were often subtle, with the bulk of the population having LI values between −0.05 and +0.05. For example, the AF showed a consistent leftward FA asymmetry with a population median LI ranging from −0.02 to −0.03 across age and 25^th^/75^th^ centiles from −0.05 to −0.01. In contrast, macrostructural features exhibited a much wider range of individual variability, often spanning the range from −0.2 to +0.2, with larger median effects (e.g., median LI for ATR length ~0.05; FPT volume ~−0.07). Second, patterns were highly dependent on the combination of tract and metric, where pathways can show opposite lateralization directions across features, highlighting the need to study both micro- and macrostructure jointly. For example, the AF shows left-lateralization for FA and volume but right-lateralization for RD and length, or the ATR showing general leftward asymmetries for diffusivities and rightward volume and length asymmetries. Third, these asymmetries were not static but changed dynamically across the lifespan, with many tracts showing age-dependent increases or decreases in lateralization. Fourth, despite these complex dynamics, male and female median trajectories showed general overlap across nearly all tracts and features, suggesting minimal sex differences in the average patterns of asymmetry. Finally, inter-individual variability was not constant, with wider centile bands in early childhood and older age suggesting a greater diversity of asymmetry patterns during these life stages.

All individual lifespan charts for all 30 tracts and 7 features are provided in the Supplementary Material (Supplementary Fig. 2).

### Which features and pathways exhibit population-level asymmetry at critical lifespan stages?

**Figure 4.**
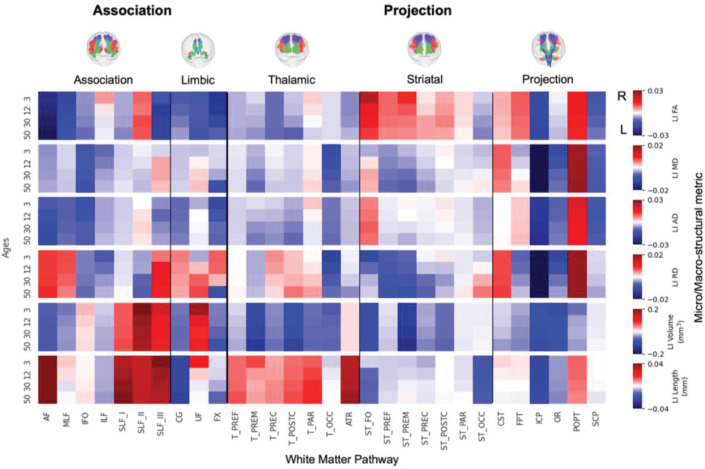
Population-level asymmetry at key lifespan milestones is highly specific to anatomical pathway and structural feature. Heatmaps depict the population-median Lateralization Index (LI) for six key features at four discrete age points (3, 12, 30, and 50 years) across 30 major white matter tracts. The color scale reflects the direction and magnitude of the median asymmetry (red: rightward; blue: leftward), highlighting that distinct patterns of lateralization are already established in early life and continue to evolve across the lifespan.

**Figure 5.**
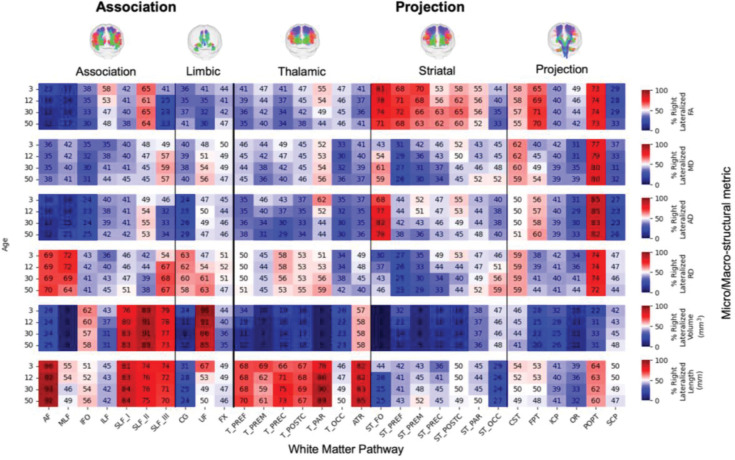
Population prevalence of rightward asymmetry across individuals. This figure complements Figure 4 by showing the percentage of the population that is right-lateralized for the same features, tracts, and age points. Numerical values and the color scale indicate this prevalence (red: >50% right-lateralized; blue: <50% right-lateralized, i.e., majority left-lateralized). This visualization reveals the consistency of asymmetry across the population, demonstrating that even for tracts with a small median LI, a strong majority of individuals may share the same direction of lateralization.

To characterize asymmetry at discrete life stages, we examined the population median LI (Figure 4) and the percentage of individuals with rightward lateralization (Figure 5) at four key ages: 3, 12, 30, and 50 years. Together, the two figures convey both the typical direction/magnitude (median LI) and the population consensus (prevalence) for each tract–feature–age combination.

The results highlight a considerable range in the degree of lateralization across the connectome. Some pathway-feature combinations showed a near-even split in directional preference across the population (i.e., median LI near 0 in Figure 4 and % right-lateralized near 50% in Figure 5). Others exhibited strong and consistent lateralization, with 80–90% or more of individuals showing asymmetry in the same direction (e.g., AF and Striato-fronto-orbital [ST_FO] volume). Importantly, many of these distinct patterns were already clearly established in early childhood (3 years old).

Examining trends by feature type revealed broad patterns. For microstructure, FA was typically left-lateralized (negative LIs) across the population for most association tracts, with the main exception being second branch of the fronto-parietal superior longitudinal fasciculus (SLF II), which tended to be right-dominant. In contrast, FA in projection tracts was more often right-lateralized in ~60–80% of the population. Other diffusivity measures (MD and AD) showed a modest but consistent leftward asymmetry across many pathways. For macrostructure, tract volume asymmetries were highly heterogeneous. Strong leftward lateralization (negative LIs) was evident for the AF (~75% of population) middle longitudinal fasiculus (MLF; ~95%), ST_FO (>95%), optic radiation (OR, ~85%), and the cingulum bundle (CG; ~80%). Strong rightward asymmetry (positive LIs) was observed for all branches (I, II, and III) of the fronto-parietal superior longitudinal fasciculus (SLF_I, 76–83%%; SLF_II, 89–91%; and SLF_III, 73–79%), and the uncinate fasciculus (UF, ~90%). Length also varied considerably; for instance, the length of AF was right-lateralized, whereas length of CG was left lateralized. Visual inspection of the heatmaps suggests these patterns are further modulated by age, a dynamic further explored in the final section.

### Do asymmetries reverse direction across the lifespan?

**Figure 6.**
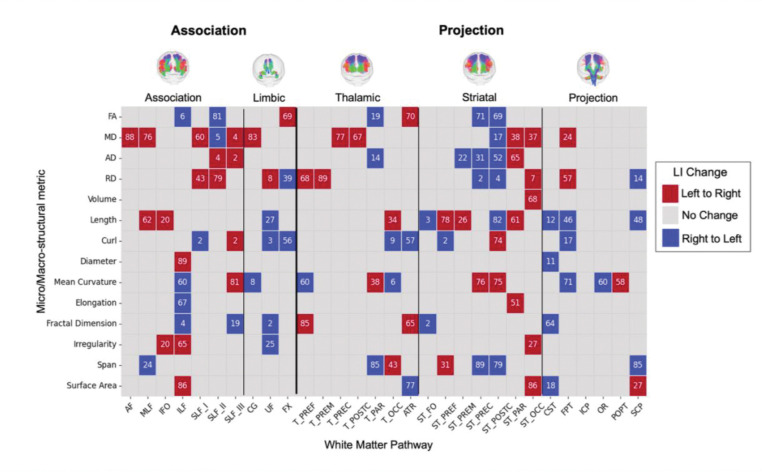
Lifespan trajectories reveal dynamic reversals in the direction of hemispheric asymmetry. Each cell indicates a “lateralization reversal,” where the population-median LI for a given tract-feature combination crosses the zero line. Red cells indicate a transition from leftward to rightward asymmetry (negative to positive LI), while blue cells indicate a transition from rightward to leftward (positive to negative LI). The overlaid numbers represent the age (in years) at which this reversal occurs. The results demonstrate that while reversals are sparse, they occur across all major pathway types and at various life stages.

Our analysis of the population median LI trajectories identified instances of “lateralization reversal,” where the typical direction of (population-level) asymmetry shifts from one hemisphere to another across the lifespan (Figure 6). This indicates that hemispheric lateralization is not a fixed characteristic but can dynamically evolve with age. As shown in the figure, reversals in the median LI, though relatively sparse, are evident across all major tract groups. There are few patterns that generalize across all pathways or all features, although we note FA tends to reverse from right-to-left (if this occurs), whereas diffusivity measures generally show the opposite left-to-right trend. The timing of these population-level transitions varies considerably across the connectome. For instance, some median LIs reversed direction early in development, while others did so much later in life. This heterogeneity suggests complex and varying degrees of hemispheric plasticity over the lifespan, likely reflecting distinct underlying neurodevelopmental and neurodegenerative processes.

### How does the magnitude of asymmetry evolve during different life stages?

**Figure 7.**
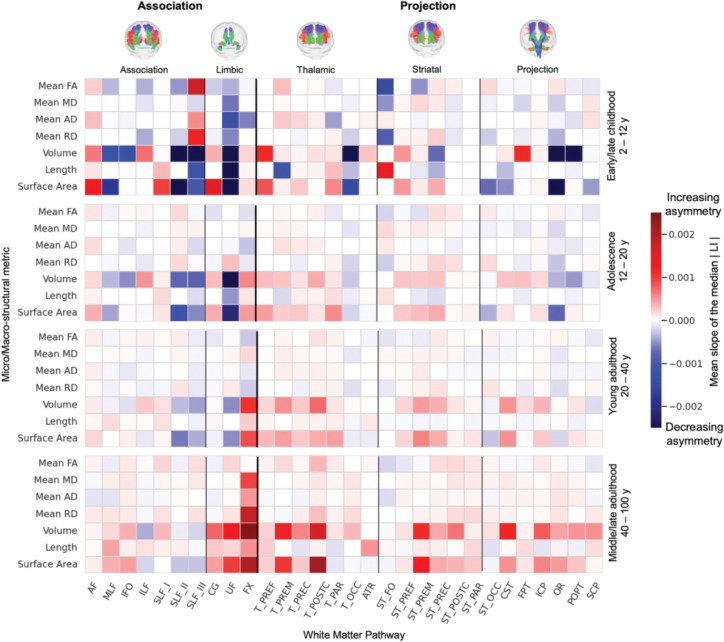
The evolution of asymmetry is a dynamic and age-specific process. This figure shows the mean rate of change (slope) of the population-median absolute lateralization index (|LI|) within four distinct life stages (rows). Each cell represents the slope for a given feature and white matter tract. Warmer colors (red) indicate an increase in the magnitude of asymmetry (strengthening of lateralization), while cooler colors (blue) denote a decrease in the magnitude of asymmetry (attenuation of lateralization). Childhood shows heterogeneous increases and decreases across pathways, adolescence largely continues these trends with smaller slopes, young adulthood is comparatively muted, and later adulthood shows a predominant increase—most marked for macrostructural measures (volume, surface area).

Finally, we quantified how the magnitude of asymmetry evolves by calculating its rate of change (the slope of the absolute value of the median LI) within four distinct life stages: early/late childhood (2–12 years), adolescence (12–20 years), young adulthood (20–40 years), and middle/late adulthood (40–100 years). Figure 7 shows these results, where an increasing asymmetry (greater |LI|, meaning *more* leftward or *more* rightward asymmetry) is indicated in red, and decreasing asymmetry (convergence towards symmetry, i.e., *less* leftward or *less* rightward) is shown in blue.

Childhood shows the most heterogenous patterns. Within the same feature class, some pathways show increasing symmetry while other show decreasing. For volume, increasing asymmetry is observed in AF, inferior longitudinal fasciculus (ILF), and several frontal/prefrontal connections (e.g., thalamo-prefrontal [T_PREF], ATR, FPT), whereas SLF-II, SLF-III, UF, and OR tend to decrease. For FA, increases appear in AF, SLF_III, and thalamo-premotor (T_PREM), while ST_FO, MLF, and ILF decrease. This mixture suggests active, pathway-specific refinement in childhood.

Adolescence largely continues the childhood directions but with smaller slopes (i.e., changes persist but are less pronounced), consistent with a tapering of developmental remodeling. Young adulthood shows mostly modest shifts, with residual increases in selected macrostructural measures (volume and surface area standing out) and relatively small changes in diffusion metrics.

In middle/late adulthood, a trend emerges where the magnitude of asymmetry generally increases across a wide range of tracts and features. This effect was particularly strong for macrostructural measures like volume and surface area, which had generally been tending towards symmetry in earlier stages of development. For example, the MLF, UF, and nearly all projection pathways showed a strengthening of asymmetry in later life. In contrast, decreases in asymmetry (i.e., tracts becoming more symmetric) were less frequent, though notable exceptions were observed (including the volume of the SLF_II and SLF_III).

## Discussion

This study represents the most comprehensive investigation of white matter asymmetry across the human lifespan. By leveraging a large-scale dataset of over 26,000 individuals from 42 neuroimaging cohorts, we generated normative lifespan trajectories for 14 distinct microstructural and macrostructural features across 30 lateralized long-range white matter pathways. Our principal findings reveal a complex and dynamic brain asymmetry across pathways, features, and the lifespan. First, we demonstrate that white matter asymmetries are widespread, affecting nearly all studied pathways, though the magnitude and lateralization vary considerably. Second, our results show that these asymmetries are feature-dependent, with different or even diverging patterns observed between measures of tissue microstructure and those reflecting pathway macrostructure. Third, we highlight that asymmetry profiles are pathway specific, likely reflecting the unique functional roles and developmental timelines of different neural pathways. Finally, we show that these asymmetries are not static but are highly dynamic, characterized by rapid, heterogeneous changes during childhood and a general trend of becoming more pronounced during the aging process. Together, these lifespan charts quantify the magnitude, direction, and age-specific variability of white-matter asymmetry, providing a reference to interpret individual differences and to study deviations in development, aging, and disease.

### Widespread White Matter Asymmetry and Comparison with Prior Literature

Our study confirms and substantially extends the view of hemispheric asymmetry as a fundamental organizing principle of the brain’s white matter connectome. By charting 30 pathways across the lifespan, we establish that asymmetries are common, yet heterogenous. A key insight from our normative modeling approach is the distinction between the *magnitude* of an asymmetry and its *consistency* across the population. While microstructural asymmetries often had modest population-median LIs, our centile charts reveal that even a small median effect can reflect a strong population consensus, with 70–90% of individuals showing the same directional asymmetry (Figure 3). Conversely, a larger median LI did not always guarantee unanimity. This highlights a critical limitation of relying on mean-based analyses alone and underscores the power of a distributional perspective for understanding brain lateralization.

These analyses also highlight that the nature and degree of asymmetry differ across both structural features and anatomical pathways, with many of these distinct lateralization patterns already evident in early infancy. Our lifespan perspective also revealed that inter-individual variability in asymmetry is not uniform (Figure 3, centile bands), with the widest centile bands observed in early childhood and in older age. This pattern likely reflects heterogeneous maturational rates and synaptic pruning during development,^41,42^ and the cumulative impact of diverse genetic, environmental, and health-related factors during aging.^43^

Compared with prior reports studying asymmetry - typically smaller, age-restricted, and focused on a few tracts or metrics (Table 2, for a review of sample size and age range see Supplementary Table 2) - our lifespan curves generally corroborate well-established findings, such as the leftward FA and volumetric asymmetry of the arcuate fasciculus in adulthood, validating our large-scale harmonization. However, our data also help clarify previously conflicting reports in the literature^44^, particularly for association pathways like the SLF, inferior fronto-occipital fasciculus (IFO), and UF. For the CST, some studies report left-lateralized microstructure,^22,45,46^ whereas others (and our results) indicate right-lateralized microstructure with left-lateralized macrostructure^45,47^ - differences that likely reflect metric choice and age window.

Several factors explain discrepancies and the additional patterns detected here. First is scale: with N = 26,199, our centile modelling estimates population trajectories rather than relying on small-sample inference. Second, continuous age modelling (rather than single time points or assumption of homogeneity across age) exposes direction changes and non-linear trends that limited-age designs miss. Third, tract definition may vary across studies.^48^ Automated, standardized segmentation (e.g., TractSeg)^31^ reduces protocol variance but will not replicate every manual or atlas-based definition used previously. Finally, is the choice of features, as micro- and macrostructural features measure different aspects of pathway biology and can often diverge. By providing comprehensive, openly available lifespan asymmetry charts, our work offers a stable reference point to help reconcile these inconsistencies and guide future research.

### Divergent Asymmetries of Microstructural and Macrostructural Features

Our findings demonstrate that white matter asymmetry is not “all encompassing” within a given pathway. Rather, it is highly feature dependent, meaning a tract broadly described as “left-lateralized” may not show leftward “dominance” for all its micro- and macrostructural characteristics. For instance, a pathway may exhibit leftward asymmetry for FA and other diffusivities (MD/AD) yet display rightward asymmetry for RD, as is seen in the arcuate fasciculus (AF) (Figure 3); or show leftward asymmetry in FA while its overall volume is greater in the right. These examples are widespread throughout our investigation (Figures 4, 5). This divergence highlights why simplistic labels such as “dominant” hemisphere for a tract or generalized statements about greater “white matter integrity” can be insufficient or even misleading without specifying which feature (reflecting distinct biological properties) is lateralized. Indeed, diffusion tensor metrics like FA and RD are sensitive to processes like myelination and axonal packing, AD to axonal structure and coherence, and MD to overall water diffusivity, each providing unique insights into pathways’ structure^16^. The field must continue to investigate the biological and functional significance of these feature-specific asymmetries to better understand brain lateralization.

### Pathway-Specific Patterns of White Matter Asymmetry

While previous work has generated such charts for grey matter^40^ and white matter,^49^ this is the first study to specifically chart asymmetry – creating population-level curves (describing the population-distribution) across age. From a basic neuroscience perspective, it provides insight into how neural circuits are lateralized in each hemisphere, lending evidence to the ongoing deduction of neuroanatomical structure-function relations.^10^

For example, established findings such as a denser, more coherent left AF (often reflected as greater FA) alongside a right-lateralized IFO volume have been interpreted as a structural basis for the left hemisphere’s efficiencies in language processing versus the right’s strengths in visuospatial integration.^50^ Further illustrating these pathway-specific patterns, our data show that the SLF complex robustly demonstrated a strong rightward volume asymmetry, with typically 70–85% of the population exhibiting this pattern across the examined ages (Figure 5); this aligns with literature noting consistent rightward volume lateralization for segments like SLF_III, linked to right-hemisphere dominant visuospatial and attentional networks.^8,44^ Moreover, such white matter asymmetries often parallel, and likely help to operationalize, established asymmetries in grey matter,^51–54^ translating asymmetric cortical regions into functionally lateralized network dynamics. Our detailed findings on numerous pathways therefore lend further credence to the body of work exploring these critical structure-function relationships. This resource of asymmetrical reference distributions can guide future research aimed at more precisely linking specific structural asymmetries to individual differences in cognitive functions, behavioral traits, and susceptibility to or presentation of neurological or psychiatric disorders.

### Dynamic Nature of Asymmetry Across the Lifespan

A central finding of this work is that white matter asymmetries are not static but follow dynamic trajectories across the lifespan (Figure 3). These dynamics are evident in both the population median LI trajectories and the corresponding population variability. While the typical magnitude of the median LI for many features, particularly microstructural ones, often remains within a relatively constrained range (e.g., frequently staying below +/−0.1), especially during most of adult life, their specific values and trajectories show population-level changes over decades. This evolution varies considerably by pathway: some tracts exhibit clear shifts and modulations in their median asymmetry profiles across developmental and aging periods (e.g. which tracts), while others maintain a more consistent pattern of lateralization and population distribution throughout much of life (e.g. AF). The periods of most pronounced change in these asymmetry trajectories - reflecting significant neural reorganization generally occur during very early development and again in later life during healthy aging. It is important to highlight the cross-sectional nature of the current dataset; because of this, we cannot (or do not) follow individual “change” but rather cross-sectional median and ranges across the population.

We also demonstrate, for the first time to our knowledge, the presence of “lateralization reversal”, where the typical direction of asymmetry for a given feature shifts across the lifespan. This phenomenon occurred in almost every tract we measured and for at least one, and often more, measures of connectivity (Figure 6), although the overall frequency was indeed sparse across pathways and features. While previous studies have demonstrated patterns of development of asymmetry over short age ranges,^55–57^ identifying specific reversal points is possible by including data across the entire lifespan. The neurodevelopmental and clinical significance of this phenomenon is, to our knowledge, completely unexplored.

Finally, our analysis reveals another novel lifespan trend: the degree of white matter asymmetry shows a general tendency to increase from mid-life to old age (Figure 7). While asymmetry in infancy/development has been a focus of prior research,^58^ its evolution during aging is less understood with limited prior investigations.^22^ Our results demonstrate that several features (volume, diameter, elongation, irregularity, and surface area) become more asymmetric with age across most pathways, and many other features show similar trends. We hypothesize that these findings are reflective of natural age-related neurodegenerative processes – occurring in individuals over 50 years old – where one hemisphere’s pathways (or specific aspects of their structure) are more vulnerable or decline at a faster rate than their contralateral counterpart, leading to the amplifying initial subtle asymmetries. Future studies are crucial to determine the neurocognitive and clinical impact, if any, of these age-related increases in white matter asymmetry.

### Functional Implications and Clinical Relevance of White Matter Asymmetry

Our findings also carry potential clinical implications. As research accumulates, white matter asymmetry measures could serve as biomarkers for certain conditions such as schizophrenia^59^ and Parkinson’s disease^27^ or as indices of typical vs. atypical brain development, such as in autism spectrum disorder.^60^ Asymmetry is a particularly attractive biomarker since it is inherently normalized to the individual via its computation as ratios or differences within the same scan. In children with neurodevelopmental disorders, it has been demonstrated that lateralization indices of diffusion metrics might serve as clinically useful imaging biomarkers, helping to detect sensory processing dysfunction with less confounding variance than absolute measures.^45^ In neurodegenerative diseases, asymmetry often manifests as an asymmetrical onset of pathology. Parkinson’s disease typically begins with unilateral motor deficits (reflecting greater degeneration of one side’s nigrostriatal pathway), and patients correspondingly show asymmetric cortical changes in several hemispheric regions^61^; a similar investigation into asymmetrical changes in white matter structural connectivity has not yet been conducted. With continued development, detection of such asymmetry measures can complement other markers to improve early detection of atypical brain development or pathology.

The role of asymmetry across disease states has only begun to be characterized. When considering clinical populations, white matter asymmetry has been a topic of intense investigation. Research in autism, for example, has observed that typical lateralization patterns are often reduced or altered. Children with autism spectrum disorer have been shown to have significantly diminished asymmetry of white matter microstructure, with many of the normal left-right differences in FA, AD, and RD being smaller in magnitude in autism spectrum disorder, indicating a more symmetrical white matter organization.^19,24^ Schizophrenia is another condition where white matter asymmetry is studied: some patients show a reduction or reversal of asymmetry in the AF and SLF, among others, with reduced asymmetry in the FA being seen in patients experiencing auditory hallucinations,^62^ possibly underlying the disturbed lateralization of language processing in schizophrenia.^24^ In such investigations of pathology-associated asymmetries, our work can serve as a cornerstone for future comparisons. By providing a multitude of tracts and measures modeled across the lifespan, our charts can reasonably be used as healthy controls in any study which seeks to investigate a particular tract, connectivity measure, and/or critical age range in an abnormal brain state. Our findings in healthy controls, therefore, potentially enables the discovery of different asymmetries that arise in pathological states, providing insight into the mechanism of a given disease.

### Limitations

Our study had notable limitations. We note the results for the fornix (FX) should be interpreted with caution as they could be a result of technical tractography artifacts rather than a biological changes; while this is particularly true of FX, such considerations can be grossly applied to all tractography methods. Macrostructural features are susceptible to the methods used to grid data and so can vary between studies. Similarly, different protocols for delineation of pathways can lead to different regions being included or excluded from a given pathway, lending further complexity to the matter of comparing study results. Lastly, there is overlap in the regions which define given pathways, meaning that microstructural measures in those regions are not unique to the given pathway.

## Conclusion

This study provides the most extensive characterization of white matter asymmetry across the human lifespan to date, generating comprehensive normative trajectories for 14 structural features across 30 distinct white matter pathways in over 26,000 healthy individuals from infancy to old age. Our key findings demonstrate that white matter asymmetries are widespread yet highly specific to pathways and structural features. Furthermore, these asymmetries are not static but exhibit dynamic changes throughout development and aging, including reversals in direction and a general trend of increasing asymmetry magnitude in later life. These lifespan charts of white matter asymmetry offer a resource for advancing our understanding of brain lateralization principles, investigating inter-individual variability in brain structure, and providing a reference to assess deviations in neurodevelopmental, aging, and clinical populations.

## Supplementary Material

Supplement 1

Supplementary Material

Supplementary figures and tables available online.

## Figures and Tables

**Table 1.** List of 30 Bilateral White Matter Tracts Included in Asymmetry Analysis.

Pathways	Subcategory	Tract	Acronym

Association	Association	Arcuate Fasciculus	AF
		Middle Longitudinal Fasciculus	MLF
		Inferior Fronto-Occipital Fasciculus	IFO
		Inferior Longitudinal Fasciculus	ILF
		Superior Longitudinal Fasciculus I	SLF_I
		Superior Longitudinal Fasciculus II	SLF_II
		Superior Longitudinal Fasciculus III	SLF_III

	Limbic	Cingulum Bundle	CG
		Uncinate Fasciculus	UF
		Fornix	FX

Projection	Thalamic	Thalamo-prefrontal	T_PREF
		Thalamo-premotor	T_PREM
		Thalamo-precentral	T_PREC
		Thalamo-postcentral	T_POSTC
		Thalamo-parietal	T_PAR
		Thalamo-occipital	T_OCC
		Anterior Thalamic Radiation	ATR

	Striatal	Striato-fronto-orbital	ST_FO
		Striato-prefrontal	ST_PREF
		Striato-premotor	ST_PREM
		Striato-precentral	ST_PREC
		Striato-postcentral	ST_POSTC
		Striato-parietal	ST_PAR
		Striato-occipital	ST_OCC

	Projection	Corticospinal Tract	CST
		Frontal Pontine Tract	FPT
		Interior Cerebellar Peduncle	ICP
		Optic Radiation	OR
		Parieto-Occipital Pontine Tract	POPT
		Superior Cerebellar Peduncle	SCP

As defined by the preset anatomical labels and segmentation outputs of the automated tractography tool TractSeg.

**Table 2.** Comparison of our findings and those previously demonstrated in the literature.

Study	Age Range	Findings	Our Findings	Study	Age Range	Findings	Our Findings	Study	Age Range	Findings	Our Findings
Amemiya et al. (2021)	6–81 years	SLF III - FA - R	✗	Parekh et al. (2023)	8–12 years	ACR – AD - R*		Thiebaut de Schotten et al. (2011)	18–22 years	CST – volume – L	✓
		SLF II - FA - R	✓			ALIC - FA - R*				CST - streamlines - L*	
		SLF I - qRI - L*				ALIC - AD - R*				OR – FA – R	✗
Budisavljević et al. (2021)	18–29 years	SLF-degree-left (right-handers)*				ALIC - MD - R*				ILF–FA–L	✓
		SLF-degree-more symmetric (left-handers)*				CG - FA - L	✓			IFOF–streamlines–R*	
Gong et al. (2005)	20–40 years	CG - FA - L	✓			CG - AD - L	✓			AF – volume – R	✗
Schmithorst et al. (2008)	5–18 years	Splenium – FA – L (girls)*				CG - MD - R	✗			AF_ant–volume–R*	
		AF – FA – R (boys)	✗			CG - RD – R	✓			AF_ant – streamlines – R*	
		Frontal WM – FA – R (boys)*				CP – FA- R*				AF_ant – FA - R*	
		Occipito-parietal WM – FA – R (boys)*				CP – AD – R*				AF_long–volume–L*	
		CST – MD – R (boys)	✓			CP – MD – R*				AF_long-streamlines-L*	
		AF – MD – R (girls)	✗			CST – FA - L	✗	Vernooij et al. (2007)	25–54 years	AF-RFD-L*	
		Occipito-parietal WM – MD – R (girls)*				CST - MD - R	✓	Wilde et al. (2009)	8–17 years	CG – FA – L	✓
Büchel et al. (2004)	21–43 years	AF–FA–L	✓			CST - RD - R	✓			Post IC – FA – L*	
		CC–FA–R*				EC - FA - L*				Thalamus – FA – L*	
		PreCG–FA–Contralateral to dominant hand	✓			EC - AD - R*				Frontal WM – FA – L (males)*	
Catani et al. (2007)	18–22 years	AF-FA-L	✓			EC - MD - R*				Frontal WM - FA – R (females)*	
Dayan at al. (2015)	5–18 years	OR-Volume-L	✓			EC - RD - R*				Ant IC – FA – L (males)*	
		OR-RD-L	✓			FX - AD - R	✗			Ant IC - FA – –R (females)*	
		OR-MD-L	✓			FX - MD - R	✗	Shu et al. (2021)	4–12 years	ATR - FA - R	✗
		OR-AD-L	✓			FX - RD - R	✓			CST - FA - R	✓
		OR-FA-L (girls > boys)	✓			ICP -FA- R*				IFOF - FA - L	✓
Demnitz et al. (2021)	62–70 years	CST-FA-L	✗			ICP - RD - L*				AF - FA - L	✓
		CST-MD-L	✗			ML - FA - L*		Dubois et al. (2016)	6–22 weeks 21–27 years	AF - FA - L (infants)*	✓
Honnedevasthana Arun et al. (2021)	22–35 years	SLF_I–FD–L*				ML - AD - R*				SLF - FA - L (infants)*	✓
		SLF_II–FD–L*				ML - MD - R*				MLF - FA - L (infants)*	✓
		SLF_IV–FD–R*				ML - RD – R*				AF - AD - L (infants)*	✓
		CG–FD–L*				PCR – AD – R*				SLF - AD - L (infants)*	✗
		IFOF–FD–R*				PCR – MD – R*				UF - AD - R (infants)*	✗
		UF–FC–R*				PCR – RD - R*				SLF - RD - R (infants)*	✓
		ILF–FD–L*				PLIC - FA - L*				MLF - RD - R (infants)*	✓
		OR–FD–L*				PLIC - AD - R*				ILF - RD - L (infants)*	✓
		ICa–FD–R*				PLIC - MD - R*				IFO - RD - L (infants)*	✓
		ICp–FD–L*				PLIC - RD - R*		Takao et al. (2013)	25 – 85 years	AF - Volume - L	✓
		SFOF–FA–R*				PTR – MD – R*	✗			CG - Volume - L	✓
		ST–FA–R*				PTR – RD - R*	✓			CC_genu - Volume - L*	
		SCR–FA–L*				RLIC - FA – L*				IC - Volume - L*	
Kumpulainen et al. (2023)	0–5 years	PTR-FA-L (infants)*	✗			RLIC – AD - R*				SLF - Volume - L*	✗
		OR-FA-L (infants)	✓			RLIC - MD - R*				EC - Volume - R*	
		PCR-FA-L (infants)*				RLIC - RD - R*				AF - FA - L	✓
		UNC-FA-R (infants)*				SCP - FA - L	✓			CG_anterior - FA - L*	✓
		PLIC-FA-R (infants)*				SCP - AD - R	✗			SLF - FA - L*	✓
		ALIC-FA-R (infants)*				SCP - MD - R	✗	Label et al. (2019)	4–33 years	AF-FA-L	✓
		SCR-FA-R (infants)*				SCP - RD - R	✗			IC-FA-L*	
		CG-FA-L (5 year-olds)	✓			SCR - FA - L*				OR-FA-L	✓
		UF-FA-L (5 year-olds)	✓			SCR - MD - R*				CG-FA-L	✓
		EC-FA-L (5 year-olds)*				SCR - RD - R*				SLF-FA-L*	✓
		SCR-FA-L (5 year-olds)*				SFO – AD – R*				SCP-FA-L	✓
		ALIC-FA-R (5 year-olds)*				SFO – MD – R*				UF-FA-L	✓
		SFOF-FA-R (5 year-olds)*				SLF - FA - L*	✓			Frontal-FA-R*	
		ACR-FA-R (5 year-olds)*				SLF - AD - R*	✗			TR-FA-R*	
Glasser and Rilling (2008)	18–50 years	AF-FA-L	✓			SLF - MD - R*	✗			Frontal-FA-L(child)-->R(adolescent)*	
Powell et al. (2006)	23–50 years	SLF-FA-L*	✓			SLF - RD - R*	✗	Baudo et al. (2022)	20–40 years	AC_anterior – fiber projections – L*	
		SLF-Volume-L*	✗			SS - AD - R*				AC_posterior – fiber projections – R*	
		IFO-FA-L	✓			SS - MD - R*		Briggs et al. (2020)	Not noted	FAT-volume-none*	
		IFO-Volume-L	✗			SS - RD - R*				IFO-volume-none	✓
		UF-FA-L	✓			UF - FA - R	✓			CG-volume-none	✗
		UF-Volume-L	✗			UF - AD - R	✗	Ford et al. (2023)	0–1 years	AF – FA – L (1–3.5 mo)*	✓
Takao et al. (2011)	21–29 years	CC_anterior-FA-L*				UF - RD - L	✗			ATR – FA – R (3–6 mo)*	✓
		CG-FA-L	✓	Putnam et al. (2010)	24–39 years	Splenium - FA - R*				UF – FA – R (0–1.5 mo)*	✗
		OR-FA-L	✓	Sboto-Frankenstein et al. (2014)	19–30 years	FX - FA - L	✓			Fx – FA – L (5–6 mo)*	✓
		SCP-FA-L	✓	Shu et al. (2015)	23.4 ± 3.7 years 12 – 35 years (using 3 standard deviations)	AF – FA – L	✓			IFO – FA – L*	✓
		IC_anterior-FA-R*				ILF – FA – L	✓			ILF – FA – R*	✓
		UF-FA-R	✗			CB – FA – L*				MI – FA – L*	
		AF_superior-FA-R*	✗			IFO – FA – L	✓			SI – FA – R*	
		AF_inferior-FA-R*	✗			CB - Fiber Number - L*				CG – FA – no asymmetry*	✗
Thiebaut de Schotten et al. (2011)	Not noted	SLF_I-Volume-None	✗			OR - Fiber Number - R*		Hau et al. (2017)	22–34 years	UF_CI – Volume – L*	✗
		SLF_II-Volume-R	✓			ILF - Fiber Number - L*				UF_C3 – volume – R*	✓
		SLF_III-Volume-R	✓			AF - Fiber Number - L*				UF_C3 – streamline count – R*	
Hasan et al. (2009)	6–68 years	UF - FA - L	✓			UF - Fiber Number - R*				UF_C5 – volume – R*	✓
		UF - AD - L	✓	Wu et al. (2016)	23–40 years	IFO - Volume - None	✓	Panesar et al. (2019)	23–35 years	vTPAT – connectivity – L*	
Label and Beaulieu (2009)	5–30 years	AF - FA - L	✓	Angstmann et al. (2016)	11–16 years	CST - FA - L	✗			dTPAT – connectivity – R*	
		AF - Streamlines - L*		Barrick et al. (2007)	20–39 years	AF - FA - L	✓			AF – volume – L	✓
Dalamagkas et al. (2020)	22–35 years	CST – FA – no asymmetry	✗	Silk et al. (2016)	10–18 years	Caudate–VLPFC – volume – R*				SLF – volume – R	✓
		HMFT – FA – R*				Caudate–DLPFC – volume – R*					

✓ = agreement between our findings and those presented in the literature. X = disagreement between our findings and those presented in the literature.

* (in “Findings”) = tract or measure not measured in our work.

* (in “Findings”) + ✓/X = tract represented in our data as conglomerate or as multiple tracts, though still allowing for gross comparison.

In cases where tract abbreviations presented in the literature differed from those of our tracts, we presented the tract name using our abbreviations.

## Data Availability

Population curves for each pathway and each feature are in format npy and available on Zenodo *[XXXXXXXXXXX].* Code for GAMLLS fitting is at https://github.com/MASILab/Asymmetry/blob/main/src/fit_model.py, code to generate figures is at https://github.com/MASILab/Asymmetry/blob/main/src/plot_fit.ipynb. Additionally, the GAMLLS models for each pathway and each feature are available on Zenodo upon publication.

## Appendix 1 Group/Consortium Authors

A) HABS-HD MPIs: Sid E O’Bryant, Kristine Yaffe, Arthur Toga, Robert Rissman, & Leigh Johnson; and the HABS-HD Investigators: Meredith Braskie, Kevin King, James R Hall, Melissa Petersen, Raymond Palmer, Robert Barber, Yonggang Shi, Fan Zhang, Rajesh Nandy, Roderick McColl, David Mason, Bradley Christian, Nicole Phillips, Stephanie Large, Joe Lee, Badri Vardarajan, Monica Rivera Mindt, Amrita Cheema, Lisa Barnes, Mark Mapstone, Annie Cohen, Amy Kind, Ozioma Okonkwo, Raul Vintimilla, Zhengyang Zhou, Michael Donohue, Rema Raman, Matthew Borzage, Michelle Mielke, Beau Ances, Ganesh Babulal, Jorge Llibre-Guerra, Carl Hill and Rocky Vig.

B) Data used in preparation of this article were obtained from the Alzheimer’s Disease Neuroimaging Initiative (ADNI) database (adni.loni.usc.edu). As such, the investigators within the ADNI contributed to the design and implementation of ADNI and/or provided data but did not participate in analysis or writing of this report. A complete listing of ADNI investigators can be found at: http://adni.loni.usc.edu/wp-content/uploads/how_to_apply/ADNI_Acknowledgement_List.pdf

C) Data used in preparation of this article were derived from BIOCARD study, supported by grant U19 – AG033655 from the National Institute on Aging. The BIOCARD study team did not participate in the analysis or writing of this report, however, they contributed to the design and implementation of the study. A listing of BIOCARD investigators can be found on the BIOCARD website (on the ‘BIOCARD Data Access Procedures’ page, ‘Acknowledgement Agreement’ document).
