# Supplementary material for: Lifespan Trajectories of Asymmetry in White Matter Tracts
Source: bioRxiv. 2025 Sep 29:2025.09.29.678806. Preprint. [Version 1] doi: 10.1101/2025.09.29.678806 (PMC12621989; doi:10.1101/2025.09.29.678806)
Supplement: Supplement 1 [file media-1.docx]

# **Supplementary Material**


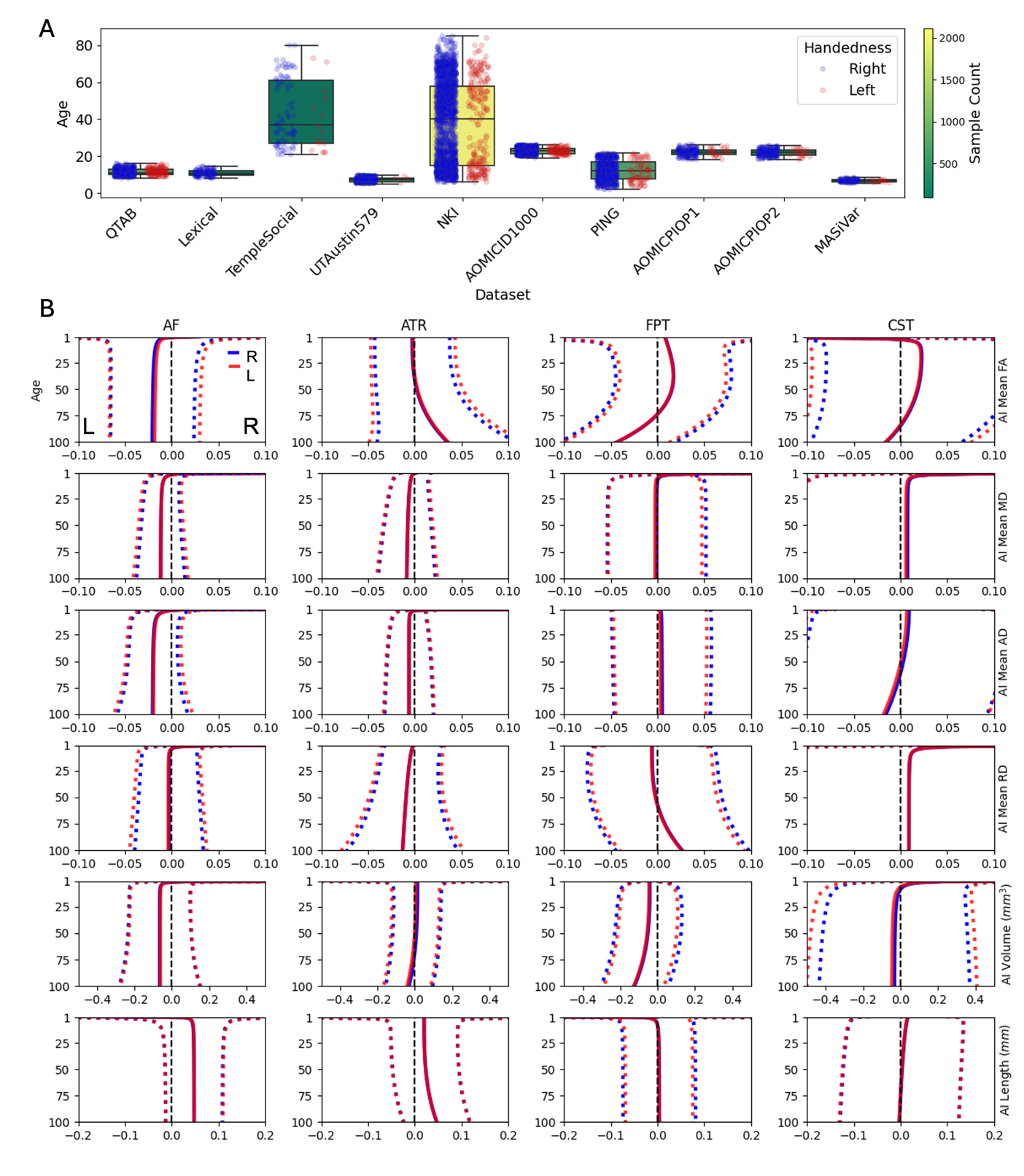


**Supplementary Figure 1: Trajectory of laterization index (LI) across age for left (L; red) and right (R; blue) handed individuals.** (A) Age distribution across datasets used in the analysis, with each dataset represented as a boxplot colored by total sample size (see colorbar). Overlaid dots indicate individual participants, colored by handedness (blue: right-handed; red: left-handed). (B) Columns correspond to white matter tracts (AF, ATR, FPT, CST), while rows represent measures including FA, MD, AD, RD, tract volume, and average tract length. Solid lines denote fitted trajectories, while dotted lines represent 95% confidence interval. A vertical dashed line at LI = 0 indicates symmetry. Positive values denote leftward asymmetry; negative values indicate rightward asymmetry. There is every little difference in handedness.


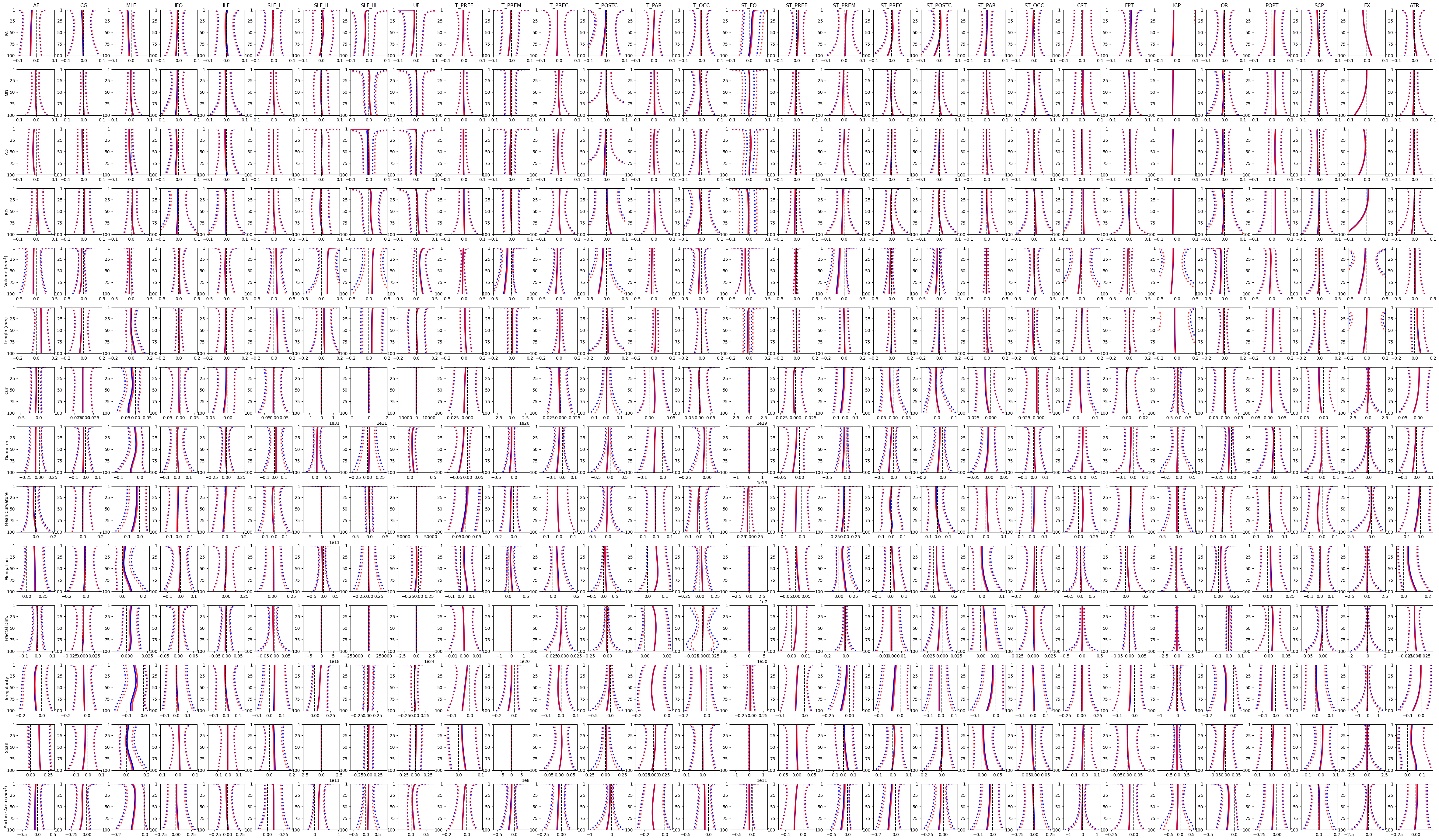


**Supplementary Figure 2: Age-related trajectories of lateralization indices across white matter tracts and diffusion/shape measures**. Each subplot displays the median (solid lines) and 95% confidence interval (dotted lines) of lateralization index (LI) across age for males (blue) and females (red). LI was modeled using the GAMLSS framework with a normal distribution family. Rows represent different microstructural and geometric measures (e.g., FA, MD, volume, curvature), while columns correspond to specific white matter tracts (e.g., AF, SLF, CST). Negative values indicate leftward asymmetry; positive values indicate rightward asymmetry. Vertical dashed lines at x = 0 highlight the zero-asymmetry point. Curves are aligned across tracts to enable direct comparison of asymmetry magnitude and lifespan trajectory by sex and measure. We observe asymmetry patterns change across tracts, measure, and age.


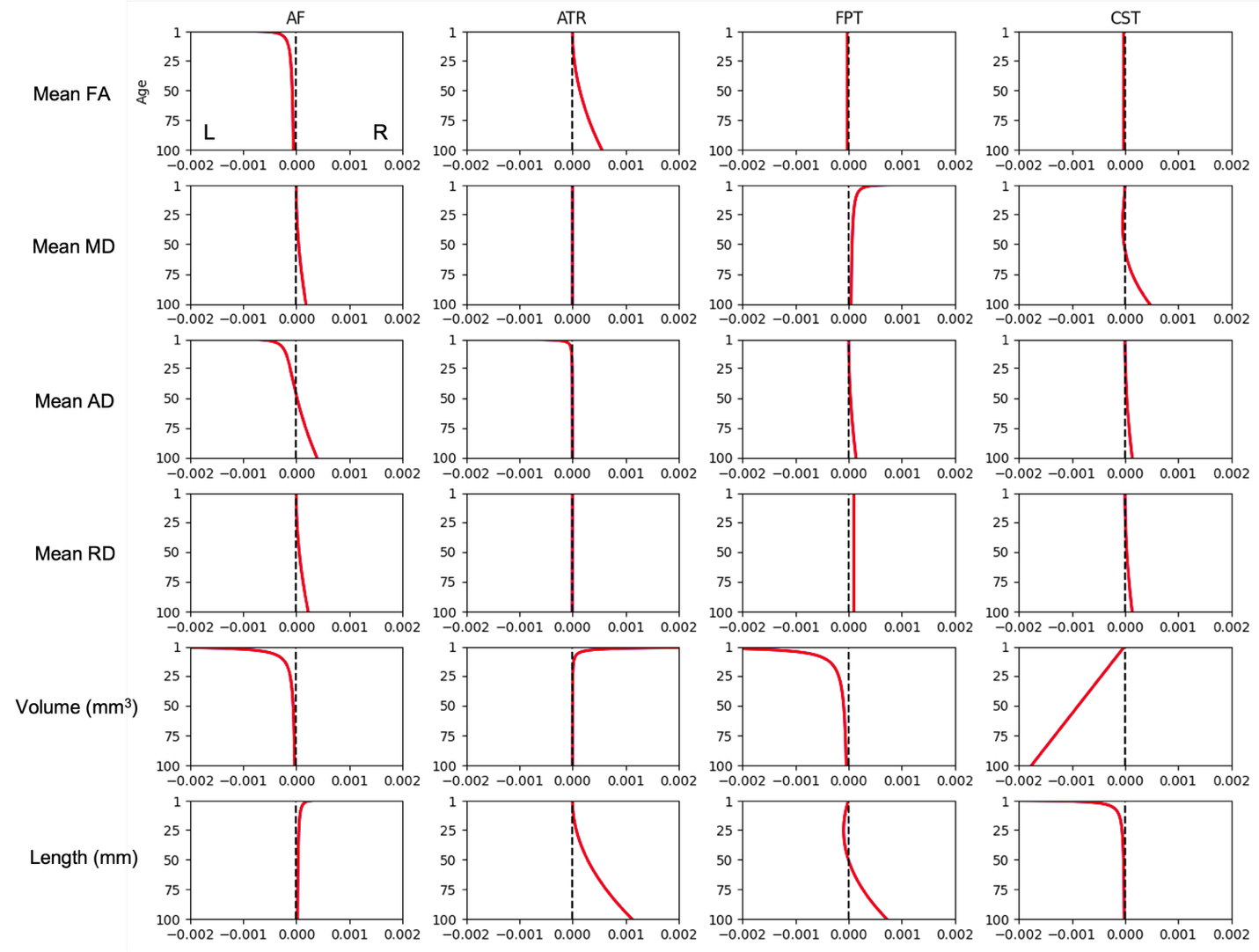


**Supplementary Figure 3. Age-related derivatives of the median LI across white matter tracts and measures.** Each subplot shows the first derivative of the 50th percentile (median) LI trajectory across age (y-axis, from 1 to 100 years) for both males (blue) and females (red) (visually hard to see as they overlap each other). Columns correspond to four major white matter tracts (AF, ATR, FPT, CST), and rows correspond to six asymmetry measures: fractional anisotropy (FA), mean diffusivity (MD), axial diffusivity (AD), radial diffusivity (RD), volume, and average streamline length. The vertical dashed line at x = 0 indicates zero change in asymmetry. Derivative curves show strong early developmental changes, especially in diffusion measures, with greater variability in structural metrics and some divergence between sexes.

**Supplementary Table 1: Overview of 42 Neuroimaging Datasets*.*** Including Subject, Session, and Scan Totals

|  | Dataset | Num Participants | Num Sessions | Num Scans | Age | %F | Country |
| --- | --- | --- | --- | --- | --- | --- | --- |
| 1 | ADNI | 1252 | 21 | 3286 | 50-95 | 51.9 | USA |
| 2 | AOMICID1000 | 888 | 0 | 888 | 19-26 | 47.5 | USA |
| 3 | AOMICPIOP1 | 204 | 0 | 204 | 18-26 | 42.6 | Amsterdam |
| 4 | AOMICPIOP2 | 224 | 0 | 224 | 18-25 | 42.9 | Amsterdam |
| 5 | BANDA | 207 | 0 | 207 | 14-17 | 35.3 | USA |
| 6 | BIOCARD | 189 | 380 | 860 | 34-91 | 35.1 | USA |
| 7 | BLSA | 1016 | 66 | 5143 | 22-103 | 44.4 | USA |
| 8 | CALM | 314 | 0 | 314 | 5-18 | 70.4 | Cambridge, UK |
| 9 | CAMCAN | 631 | 0 | 631 | 18-88 | 49.3 | Cambridge, UK |
| 10 | CUTTING | 632 | 1229 | 1511 | 4-19 | 50.3 | USA |
| 11 | Calgary | 88 | 248 | 248 | 1-6 | 53.2 | Canada |
| 12 | DLBS | 460 | 3 | 935 | 21-97 | 36.9 | USA |
| 13 | HABSHD | 2607 | 3 | 3250 | 50-92 | 37.1 | USA |
| 14 | HBN | 1955 | 0 | 1955 | 5-21 | 36.2 | USA |
| 15 | HCP | 1064 | 0 | 1108 | 22-37 | 45.4 | USA |
| 16 | HCPA | 718 | 0 | 718 | 36-100 | 43.9 | USA |
| 17 | HCPBaby | 197 | 336 | 377 | 0-10 | 48.5 | USA |
| 18 | HCPD | 621 | 0 | 621 | 5-21 | 45.9 | USA |
| 19 | Humphreys | 97 | 3 | 119 | 0-1 | 63.9 | USA |
| 20 | IBIS | 224 | 3 | 329 | 0-2 | 64.8 | USA |
| 21 | ICBM | 185 | 0 | 254 | 19-80 | 42.9 | USA Canada |
| 22 | Lexical | 113 | 1 | 113 | 8-14 | 54.9 | USA |
| 23 | MAP | 408 | 22 | 936 | 58-100 | 23.2 | USA |
| 24 | MARS | 174 | 18 | 326 | 63-95 | 13.2 | USA |
| 25 | MASiVar | 83 | 3 | 118 | 5-8 | 59.3 | USA |
| 26 | MORGAN | 255 | 7 | 413 | 17-71 | 45.5 | USA |
| 27 | NACC | 687 | 10 | 766 | 43-100 | 38.8 | USA |
| 28 | NKI | 1277 | 7 | 2167 | 6-85 | 40.9 | USA |
| 29 | PING | 697 | 0 | 711 | 2-21 | 51.5 | USA |
| 30 | QTAB | 414 | 2 | 716 | 8-16 | 50.0 | Australia |
| 31 | ROS | 47 | 24 | 79 | 62-91 | 7.6 | USA |
| 32 | SCAN | 265 | 237 | 279 | 42-93 | 38.4 | USA |
| 33 | SWU | 231 | 2 | 426 | 17-27 | 53.5 | China |
| 34 | TempleSocial | 103 | 0 | 103 | 21-80 | 36.9 | USA |
| 35 | UCLA_LA5c | 250 | 0 | 250 | 21-50 | 56.8 | USA |
| 36 | UKBB | 9116 | 0 | 9116 | 46-82 | 45.9 | UK |
| 37 | UPennRisk | 151 | 2 | 248 | 18-34 | 57.7 | USA |
| 38 | UTAustin579 | 238 | 3 | 373 | 5-9 | 45.0 | USA |
| 39 | VMAP_2.0 | 268 | 268 | 268 | 50-90 | 40.3 | USA |
| 40 | VMAP_JEFFERSON | 315 | 955 | 966 | 60-96 | 59.2 | USA |
| 41 | VMAP_TAP | 164 | 165 | 165 | 55-86 | 43.0 | USA |
| 42 | WRAP | 342 | 5 | 557 | 44-76 | 35.2 | USA |

**Supplementary Table 2. Review of studies investigating white matter asymmetry at various points in the lifespan*.*** The study method, micro- and macro-structural features investigated, number of participants, age range, and key findings are summarized. ^8,17,18,20-23,26,45-47,50,55,57,58,63-83^
